# Infection with the SARS-CoV-2 Omicron variant in children with congenital heart disease: A case series study during Shanghai epidemic

**DOI:** 10.3389/fcvm.2022.1001780

**Published:** 2022-10-11

**Authors:** Yinyu Yang, Yibei Wu, Wen Zhang, Qing Cao, Haibo Zhang, Hao Zhang, Wei Dong

**Affiliations:** ^1^Department of Cardiothoracic Surgery, Shanghai Children's Medical Center, Shanghai Jiaotong University School of Medicine, Shanghai, China; ^2^Department of Infectious Disease, Shanghai Children's Medical Center, Shanghai Jiaotong University School of Medicine, Shanghai, China

**Keywords:** children, congenital heart disease, coronavirus, Omicron, infectious disease

## Abstract

**Objective:**

To analyze the clinical characteristics and prognostic factors of severe acute respiratory syndrome coronavirus 2 (SARS-CoV-2) Omicron variant infections in children with congenital heart disease (CHD).

**Methods:**

A retrospective analysis was performed on SARS-CoV-2 Omicron-infected children with CHD who were admitted to Shanghai Children's Medical Center from April 1, 2022 to May 31, 2022. The clinical, laboratory and imaging data, and the nucleic acid conversion time of the children in this group were collected and analyzed.

**Results:**

Thirteen patients were included in this study and had an average age of 1.1 (0.16–14) years. Among the patients, 3 patients were preoperatively treated, and 10 were postoperatively treated. According to the severity of the disease, 1 patient was diagnosed with the moderate type, and the remaining 12 patients were diagnosed with the mild type. The clinical symptoms were mostly associated with upper respiratory tract infections, including 13 with fever (100%), 8 with cough (61.8%), 5 with sputum production (38.5%), 1 of shortness of breath (7.7%), etc. All patients were successfully discharged from the hospital, with 16.4 ± 2.9 days needed to obtain cycle threshold (CT) values ≥35 in nucleic acid testing and 17.5 ± 3.6 days of hospitalization.

**Conclusions:**

For vulnerable patients such as children with CHD, SARS-CoV-2 Omicron variant infections mostly present with mild upper respiratory tract symptoms with negative or mildly changed chest imaging. Through appropriate treatment of the underlying disease in the quarantine ward, patients might obtain good outcomes, even after long periods of hospitalization.

## Introduction

As of May 1, 2022, there have been more than 500 million people infected with severe acute respiratory syndrome coronavirus 2 (SARS-CoV-2) and 6 million deaths worldwide (1). On November 9, 2021, a new variant of SARS-CoV-2, B.1.1529, was first detected in South Africa, and the World Health Organization (WHO) named it the Omicron variant on November 26, 2021 (2–4). Due to the high transmissibility of the omicron variant, a new wave of global epidemics has occurred over the last several months (5–9). Moreover, a large number of pediatric patients with omicron variant infections were detected (10–12).

In late February 2022, a wave of SARS-CoV-2 infection rapidly appeared in Shanghai as of May 4, 2022, and 601,942 patients were diagnosed with SARS-CoV-2 infections, including 503 deaths. All the new viral genomes in Shanghai were clustered into the SARS-CoV-2 BA.2.2 sublineage (13). Patients under 18 years old, including several patients with congenital heart disease (CHD), were admitted to the quarantine ward in Shanghai Children's Medical Center affiliated with Shanghai Jiaotong University School of Medicine during this outbreak in Shanghai. To the authors' knowledge, there have been no reports of Omicron variant infections among the pediatric CHD population. We presented the first single-center case series study of the clinical features of CHD children with Omicron infections.

## Materials and methods

From April 1, 2022, to May 31, 2022, in our center, all patients younger than 18 years old who were positive for SARS-CoV-2 nucleic acid test by pharyngeal/nasal swab (fluorescence quantitative polymerase chain reaction (PCR) method, cycle threshold (CT) cut-off value 43) were reviewed, and patients with CHD were included in this study. The hospital ethics committee approved this study and waived the need for individual consent (SCMCIRB-K2022053-3). During hospitalization, the patients were classified according to the Chinese Health Commission's Novel Coronavirus Pneumonia Diagnosis and Treatment Program (Version 9) (14): mild, moderate, and severe. The discharge criteria (14) were as follows: (1) The patients' temperature had normalized for more than 3 days; (2) the respiratory symptoms had improved significantly; (3) chest imaging showed significant improvement; (4) a CT value ≥35 was noted for the N gene and ORF gene on two consecutive nucleic acid tests (based on the fluorescence quantitative PCR method with a threshold value of 40 and if the sampling interval was >24 h) or negative nucleic acid results on two consecutive tests (based on the fluorescence quantitative PCR method with a threshold value of 35 and if the sampling interval was >24 h).

All the basic characteristics, clinical symptoms, laboratory examinations and chest imaging of the study patients were collected and analyzed by clinical staff. The clinical symptoms mainly include fever, cough, sputum, shortness of breath, etc. The laboratory examinations mainly included routine blood examinations at the time of admission and CT values of the pharyngeal/nasal nucleic acid N gene each time during the outpatient period/admission period. The imaging examinations were the chest X-rays at admission.

All data in this study were statistically analyzed with SPSS 25.0 software. A line chart was used to describe the time trend of the CT value in patients. Categorical data are expressed as the number of cases (percentage %). Measurement data with a normal distribution are expressed as the mean ± standard deviation, and measurement data with an abnormal distribution are expressed as the median (range). Comparison between two groups of continuous variables with normal distribution was made using student's *t*-test. *P* < 0.05 was considered statistically significant.

## Results

This study reviewed a total of 625 patients admitted to the quarantine ward in our center from April 1, 2022, to May 31, 2022. Finally, 71 patients (11.36%) had pre-existing medical conditions. Thirteen (2.08%) CHD patients were included in this study. Median age was 1.1 (0.16–14) years, and mean weight was 11.03 ± 5.59 kg. None of the children in our study were previously vaccinated. The clinical features of the 13 patients are listed in Table 1.

**Table 1:** Clinical features of the patients with congenital heart disease.

**Basic status**	***N***
Sex, male%	5 (38.5%)
Age, y	1.1 (0.77,3.75)
Weight, kg	11.03 ± 5.59
**Severity of the disease**
Mild	12
Moderate	1
**Presenting features**
Fever	13 (100%)
Cough	8 (61.8%)
Sputum	5 (38.5%)
Short of breath	1 (7.7%)
Others (e.g., diarrhea, seizures, and headache)	0
**Laboratory results (on admission)**
Hemoglobin, g/dL	12.11 ± 2.46
Platelets, 10^9^/L	243.75 ± 115.30
White cell count, 10^9^/L	7.18 ± 3.06
Neutrophil, %	47.69 ± 18.19
Lymphocyte, %	41.57 ± 21.15
C-reactive protein, mg/L	6.94 ± 6.03
Discharged, %	13 (100%)
Hospital stay, days	17.5 ± 3.6
Time of CT value>35 in nucleic acid, days	16.4 ± 2.9

CT, cycle threshold.

Among the 13 children with CHD, 3 patients were preoperatively diagnosed: 2 were diagnosed with ventricular septal defects (VSD), and 1 was diagnosed with pulmonary atresia with VSD (PA/VSD). Ten patients were postoperative: 3 patients had VSD, 2 had an atrial septal defect (ASD), 1 had severe mitral regurgitation (MR), 3 had pulmonary artery sling (PAS) with tracheal stenosis, and 1 had tetralogy of Fallot (TOF). The time from operation to SARS-CoV-2 infection was 3.5 (0.5–114) months. Details of all 13 patients are shown in Table 2.

**Table 2:** Clinical and paraclinical findings of the patients with congenital heart disease.

**Patient no**.	**Age (y)**	**Type of congenital heart disease**	**Surgical repair**	**Interval between surgery and infection (months)**	**Signs and symptoms**	**Administered drugs**	**Chest X-ray**	**Hospital stay (d)**	**Adjuvant therapy**
1	14	MR (severe)	Yes	1.5	Fever	Ibuprofen, LQC	Negative	13	
2	3	PAS/CTS	Yes	0.5	Fever, cough, sputum	Ibuprofen, LQC, ambroxol	Obvious exudation	14	Nebulization and occupational therapy
3	0.77	VSD, ASD, TR(moderate), Post-ECMO	Yes	4	Fever, cough, sputum	Ibuprofen, LQC, ambroxol	Mild change	15	
4	0.7	PAS/CTS	Yes	0.6	Fever, cough, sputum	Ibuprofen, LQC, ambroxol	Mild change	16	Nebulization and occupational therapy
5	1	PAS/CTS	Yes	0.5	Fever, cough, sputum	Ibuprofen, LQC, ambroxol	Mild change	19	Nebulization and occupational therapy
6	1.1	VSD/PH	Yes	9	Fever, cough	Ibuprofen, LQC	Negative	21	
7	3.75	ASD	Yes	5.5	Fever	Ibuprofen, LQC	Negative	19	
8	1.25	ASD	Yes	3	Fever	Ibuprofen, LQC	Negative	19	
9	1.1	TOF	Yes	9	Fever, cough	Ibuprofen, LQC	Negative	24	
10	0.16	VSD	No	/	Fever	Ibuprofen, LQC	Mild change	23	
11	12	VSD/PH	Yes	114	Fever, cough, sputum, short of breath	Ibuprofen, LQC, ambroxol, furosemide, digoxin	Obvious exudation	16	Mask oxygen
12	0.4	VSD/PH	No	/	Fever, cough	Ibuprofen, LQC	Mild change	14	
13	6.8	PA/VSD	No	/	Fever	Ibuprofen, LQC	Mild change	15	

ASD, atrial septal defect; CTS, congenital tracheal stenosis; ECMO, extracorporeal membrane oxygenation; LQC, Lianhua Qingwen capsule; MR, mitral valve regurgitation; PA, pulmonary atresia; PAS, pulmonary artery sling; PH, pulmonary hypertension; TOF, tetralogy of Fallot; VSD, ventricular septal defect.

According to the severity of the disease, our study included 1 moderate patient and 12 mild patients. The moderate patient (patient 11) underwent VSD surgery 9 years ago. He was admitted to the hospital due to high fever and shortness of breath during this episode. The chest X-ray showed bilateral lung exudation. The patient was given oxygen inhalation with a mask during the first 5 days. Digoxin was used to strengthen the cardiac output, and furosemide was used for diuresis. The patient was successfully discharged after 20 days of hospitalization without the need for mechanical ventilation. Three mild patients (patients 2/4/5) underwent slide tracheoplasty while correcting the cardiac anatomy. These three patients were treated with nebulization and occupational therapy while they were in the quarantine ward to prevent airway obstruction due to excessive secretions. There was a special mild case (patient 3) who unfortunately suffered cardiac arrest after VSD repair and was saved by ECMO in the intensive care unit. Then, a second intervention was performed during ECMO support. Residual moderate tricuspid regurgitation and a 3 mm ASD were reserved. The patient was admitted to the quarantine ward after having been confirmed positive on the nucleic acid test, and the patient had residual anatomical problems. However, beyond our expectations, the patient was quite stable during the hospital stay and did not require any additional treatment.

The results of routine blood tests in the 13 patients admitted to the hospital are shown in Table 1. The results of chest X-ray examination are shown in Table 2. Obvious exudation was detected in only two patients, and mild or no change was detected in the remaining patients. No specific electrocardiographic or echocardiographic changes related to the infection were detected.

**Figure 1:**
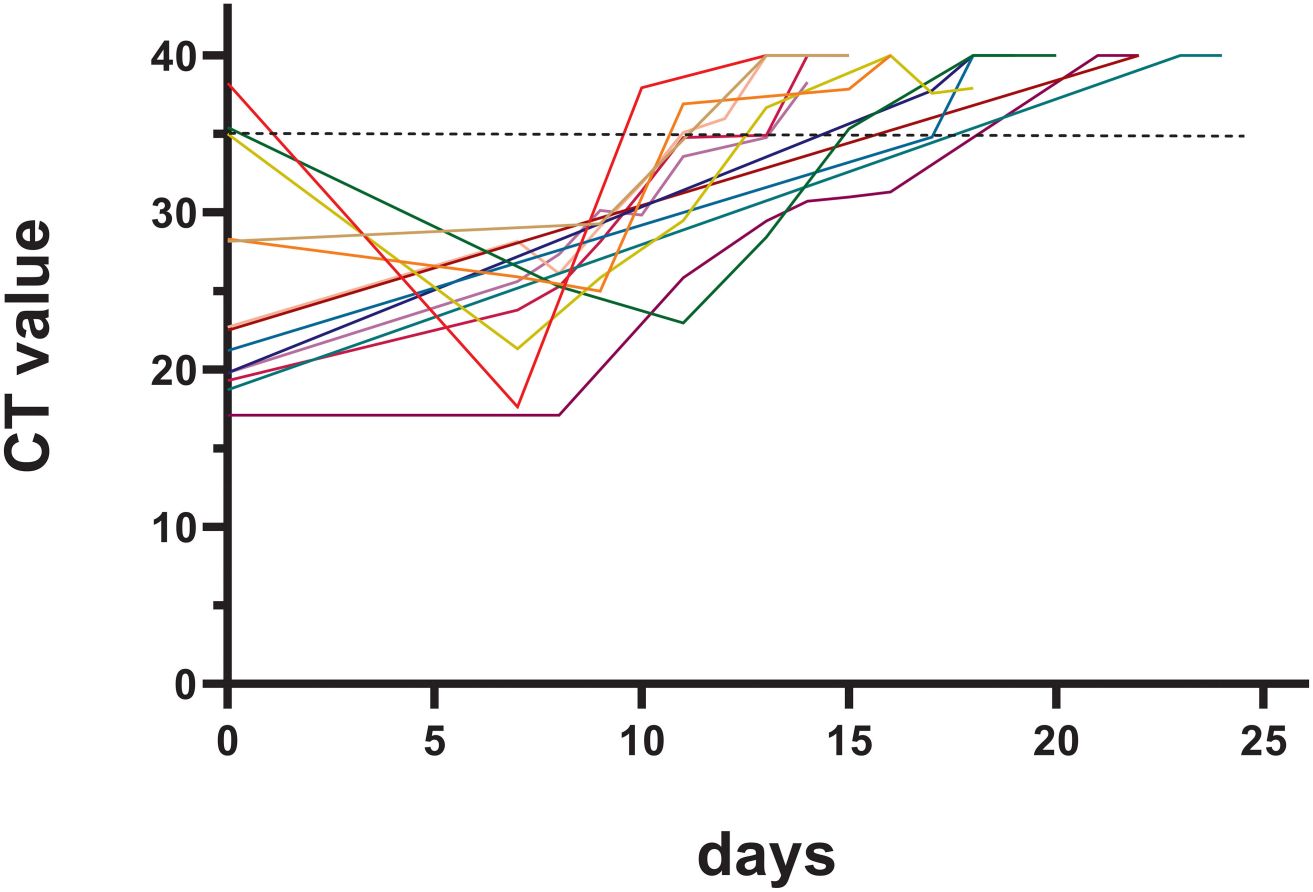
Changes in the cycle threshold (CT) value of nucleic acid tests.

The pharyngeal/nasal swab nucleic acid test was an important laboratory test during hospitalization. The basic CT value of the nucleic acid N gene of the patients in this study was 25.09 ± 6.10, and it took 16.4 ± 2.9 days to meet the basic criteria for discharge. The duration of viral clearance was similar between patients infected during the early postoperative period (within 1 month after surgery) and the late postoperative period (15.0 ± 1.7 days vs. 16.7 ± 4.2 days, *p* = 0.528). The changes in the CT value of the nucleic acid N gene are shown in Figure 1.

All these patients were free of symptoms during the follow-up period. The three preoperatively diagnosed patients had regular clinic visit and the operations were scheduled in the near future.

## Discussion

Recently, the SARS-CoV-2 Omicron variant has become the most prevalent variant in the world. Several studies have also found that children have been susceptible to the Omicron variant in the last 6 months (10–12). There is no doubt that children with CHD are a special category in the pediatric population. We reported a series of 13 cases of pediatric patients with Omicron variant infections and congenital heart disease that have not been reported in previous studies. All patients were successfully discharged with no complications.

Since the pandemic of SARS-CoV-2 in 2020, children with CHD have been a vulnerable group for this type of disease. The severity, complications, and mortality of pediatric patients with CHD were higher than those without CHD. In an early study in 2020 (15), 53 CHD patients were reported to be infected with SARS-CoV-2. Ten patients were children (2 moderate-severe cases), and there was a total of 3 (6%) deaths. In a retrospective study by Strah et al. (16) in 2021, a total of 160 cases of CHD pediatric patients were included. The patients with CHD were younger (1 vs. 11 years), had a longer length of stay (22 vs. 6 days), had a higher complication rate (6.9 vs. 1.1%), had higher mortality rates (3.8, 0.8%), and had higher costs ($54,619 vs. 10,731; *p* < 0.001 for all). Moreover, in a recently published study comparing children with CHD and non-CHD patients, it was found that the pediatric patients with CHD had a higher ICU admission rate (55.6% vs. 31.9%) and higher use of mechanical ventilation (18.5% vs. 6.5%).

As the first study on CHD combined with the omicron variant, there were no severe cases in our study, and all 13 patients were successfully discharged. This might be because the omicron variant was thought to be less severe than the former variants in terms of the disease severity (17). As was first reported regarding the Omicron prevalence in children in South Africa (10), seven (5%) of 138 children were ventilated, and four (3%) died during the study period. All deaths were related to complex underlying diseases. Therefore, CHD, as one of the most common underlying diseases in children, still needs to be given special attention by clinical staff. In our study, our medical team in the quarantine ward included skilled CHD surgeons, intensive care unit specialists and experienced nurses who were very familiar with the pathophysiology of different types of CHD. Proper management of underlying diseases might be one of the factors that contributed to the successful discharge of all our patients in this study.

At present, the vulnerable factors of Omicron infection in children with CHD are described as follows:

First, the characteristics of the Omicron variant were reported in recent studies (6): due to the extensive spike protein mutation, which plays an essential role in SARS-CoV-2 evolution via changes in receptor-binding domain and neutralizing antibody epitope presentation, the omicron variant had high levels of immune evasion and transmissibility. Furthermore, the Omicron receptor-binding domain increased ACE2 (host receptor) binding, which then worsened the SARS-CoV-2 transmission and disease severity (18). The properties of these structures have led to the quick spread of Omicron variants around the world.

Another influencing factor was vaccination status. In adult studies, it was found that the 3 doses of vaccine provided a good protective effect against Omicron infection. In a recent study of pediatric patients (19), the estimated vaccine effectiveness for 2 doses of BNT162b2 against symptomatic infection decreased rapidly (60.1 to 28.9% in children and 59.9 to 16.6% in adolescents), and among adolescents, it increased after a booster dose (71.1%). However, patients with CHD might be younger than non-CHD patients when infected. Due to the current policy, children with symptomatic CHD start getting vaccinated against coronavirus disease 2019 (COVID-19) 6 months postoperation. They did not receive any sort of SARS-CoV-2 vaccine. Therefore, the development of effective and safe vaccines for patients of younger ages and special populations is an urgent problem that needs to be solved.

From the perspective of children with CHD, there were also factors that made them more susceptible to infection than non-CHD patients. For children who are being treated prior to CHD surgery, some studies believe that (20) CHD-induced congestive heart failure (left-to-right shunt) or cyanosis (right-to-left shunt) might lead to chronic inflammatory responses. Moreover, the influence of SARS-CoV-2 resulted in further immune activation and failure of some lymphocyte subsets. It requires special attention, as CHD could represent a population particularly at risk during the COVID-19 pandemic.

For postoperative children, the whole or part of the thymus needs to be removed during the process to ensure the surgical field of view since most CHD surgeries require the midsternotomy approach. However, premature removal of the thymus at a young age could also lead to abnormal T-cell differentiation. In a long-term follow-up study (21), thymectomized CHD patients more frequently developed cancer, autoimmune diseases, and atopic diseases and had a higher risk for viral infections (59.5 vs. 19.9%, *p* < 0.001). In this study, among the 10 postoperative children, all required a midsternotomy approach in cardiac surgery, and the possibility of removal of the thymus during the operation was high, which might be one of the factors why they were more likely to be infected by the Omicron variant. However, with the continuous advancement of surgical techniques, many CHDs can be treated surgically with the minimally invasive operation technique. They can be treated with a subaxillary incision, which could significantly reduce the proportion of surgery-related thymectomies.

Another possible factor might be cardiopulmonary bypass (CPB). Most CHD surgeries still need to be performed with CPB. However, early studies pointed out that the severe systemic inflammatory response caused by CPB (22, 23) often leads to the disturbance of immune function in the short term after surgery. In a case series study of patients with SARS-CoV-2 infections after cardiac surgery (24), the effect of CPB on the inflammatory system was regarded as a confounding factor of SARS-CoV-2 infection, and it requires special attention. All the postoperative patients underwent CPB during surgical repair in our study. Perhaps this might be one reason why the patients were infected.

In addition, some of the children with CHD had genetic abnormalities, the most common being Down syndrome, DiGeorge syndrome and Noonan syndrome. These genetic defects were associated with immune deficiencies, which may increase the risk of Omicron infection and the occurrence of severe diseases.

Another finding in our study was that it took a long hospital time (16.4 ± 2.9 days) for CHD patients to improve to meet the discharge standard, which was not reported in previous studies. A longer duration of infection might increase the risk of the patient developing severe disease. Therefore, one question to ask is how can we shorten the required time to reach the adequate CT value to prevent mild cases from developing into severe or critical cases? The baseline CT value in some of our patients was above 35. These patients were in the incubation period or early stages of the disease, and a decreased CT value could be found on Day 7 (Figure 1) in these patients.

Since 2020, the SARS-CoV-2 pandemic has affected cardiac surgery to varying degrees (25, 26). The operation time of preoperative patients was delayed. Some children with severe heart diseases lost their indications for surgery and died. In our study, 3 patients were preoperatively enrolled. They were able to meet the discharge criteria at 22, 11, and 14 days. At present, there are several reports on the treatment of critically ill CHD after negative nucleic acid testing for SARS-CoV-2 (27) and surgical treatment in the recovery period for SARS-CoV-2 (28). These studies have a positive effect on exploring the reasonable timing of surgery in children with CHD during the SARS-CoV-2 outbreak. As clinical staff are working on CHD disease patients, we should increase our attention to this type of research.

### Limitations

This study had several limitations. First, our study was a single-center retrospective study with a limited number of samples and a short study time, and was limited by the selection bias. Second, the main criterion for discharge in this study was based on the CT value of the N gene on the nasopharyngeal nucleic acid test. However, in actual work, we found some sampling bias in the CT value results caused by the sampling techniques of nasopharyngeal swabs. Third, the sampling time of the nucleic acid in our study started from the 7th day after admission. We did not conduct nucleic acid sampling within 7 days because of the following reasons: (1) the possibility of children's CT value reaching the discharge standard was extremely low; (2) due to the large number of patients in the ward, it was reasonable to reduce the number of samplings in order to reduce the risk of medical staff being infected. Such a protocol inevitably led to a certain deviation in the nucleic acid CT value curve.

## Conclusion

Children with CHD are at risk of infection with the Omicron variant. As the first report in CHD children with Omicron, none of the patients in our study became severe cases, and careful treatment of the underlying diseases might lead to successful discharge. Although the Omicron variant might be less serious, it took a relatively long time to meet the discharge criteria.

## Data availability statement

The raw data supporting the conclusions of this article will be made available by the authors, without undue reservation.

## Ethics statement

The studies involving human participants were reviewed and approved by Shanghai Children's Medical Center. Written informed consent from the participants' legal guardian/next of kin was not required to participate in this study in accordance with the national legislation and the institutional requirements. Written informed consent was not obtained from the minor(s)‘ legal guardian/next of kin for the publication of any potentially identifiable images or data included in this article.

## Author contributions

YY, YW, and WZ were responsible for conceptualization, data curation, and writing the manuscript. QC was responsible for data curation and statistical analysis. HaiZ, HaoZ, and WD were responsible for conceptualization, reviewing, editing, and supervision. All authors contributed to the article and approved the submitted version.

## Funding

This study was supported by Shanghai Municipal Science and Technology Commission Research Project (19411950200), Shanghai Hospital Developing Center (SHDC12018128), and Key Discipline Group Development Fund of Health and Family Planning Commission of Pudong New District (PWZxq2017-14).

## Conflict of interest

The authors declare that the research was conducted in the absence of any commercial or financial relationships that could be construed as a potential conflict of interest.

## Publisher's note

All claims expressed in this article are solely those of the authors and do not necessarily represent those of their affiliated organizations, or those of the publisher, the editors and the reviewers. Any product that may be evaluated in this article, or claim that may be made by its manufacturer, is not guaranteed or endorsed by the publisher.
